# Supplementary material for: Timing of Vertical Head, Withers and Pelvis Movements Relative to the Footfalls in Different Equine Gaits and Breeds
Source: Animals (Basel). 2022 Nov 7;12(21):3053. doi: 10.3390/ani12213053 (PMC9657284; doi:10.3390/ani12213053)
Supplement: Supplementary file 1 [file animals-12-03053-s001.zip › animals-1860504-supplementary.pdf]

Supplementary Table 1

Model results for stride duration at walk and trot, diagonal dissociation and suspension at trot are presented as estimated marginal means (EMM) and lower/upper confidence intervals (CI). Significant pairwise comparisons between breeds (WB= Warmbloods) are presented at the bottom of the tables.

	**Suspension - Trot**		
	**EMMs**	**Lower C.I**	**Upper C.I**
Iberian	2.99	1.11	4.86
Icelandic In Hand	6.07	4.19	7.94
Icelandic Ridden	2.55	0.82	4.27
WB	5.75	3.68	7.82
Between-breed significant pairwise comparison ($p<0.005$)	Iberian – Icelandic In Hand<br>Icelandic Ridden – Icelandic In Hand<br>WB– Icelandic Ridden		

	**Stride duration(s) - Walk**		
	**EMMs**	**Lower C.I**	**Upper C.I**
Iberian	1.2	1.16	1.26
Icelandic In Hand	1.04	0.98	1.1
Icelandic Ridden	1	0.95	1.05
WB	1.3	1.24	1.35
Between-breed significant pairwise comparison ($p<0.001$)	Iberian – Icelandic (Ridden and In Hand)<br>WB - Icelandic (Ridden and In Hand)		

	**Stride duration (s) - Trot**		
	**EMMs**	**Lower C.I**	**Upper C.I**
Iberian	0.76	0.71	0.76
Icelandic In Hand	0.64	0.61	0.66
Icelandic Ridden	0.58	0.56	0.6
WB	0.78	0.75	0.79
Between-breed significant pairwise comparison ($p<0.001$)	Iberian – Icelandic (Ridden and In Hand)<br>WB - Icelandic (Ridden and In Hand)<br>Icelandic Ridden – Icelandic In Hand		

	**Diagonal dissociation - Trot**		
	**EMMs**	**Lower C.I**	**Upper C.I**
Iberian	-1.15	-2.55	0.24
Icelandic In Hand	-4.59	-6.29	-2.88
Icelandic Ridden	-5.73	-7.16	-4.31
WB	-3.5	-5.04	-1.93
Between-breed significant pairwise comparison ($p<0.005$)	Iberian – Icelandic (Ridden and In Hand)<br>WB – Icelandic (Ridden and In Hand)<br>Iberian – WB		
